# *Eugenia uniflora* Effects on the Depressive-like Behavior of MPTP-Exposed Female Rats: Apoptosis and α-Synuclein Modulation

**DOI:** 10.3390/brainsci15010041

**Published:** 2025-01-03

**Authors:** Anne Suély Pinto Savall, Jhuly Dorneles De Mello, Eduarda Monteiro Fidelis, Vandreza Cardoso Bortolotto, Mustafa Munir Mustafa Dahleh, Gustavo Petri Guerra, Marina Prigol, Robson Puntel, Jean Ramos Boldori, Cristiane Casagrande Denardin, Tuane Bazanella Sampaio, Simone Pinton

**Affiliations:** 1 Research Group on Biochemistry and Toxicology in Eukaryotes, Federal University of Pampa—Campus Uruguaiana, Uruguaiana 97500-970, RS, Brazil; annesavall@gmail.com (A.S.P.S.); eduardamonfil.aqua@gmail.com (E.M.F.);; 2 Laboratory of Pharmacological and Toxicological Evaluations Applied to Bioactive Molecules (LaftamBio), Federal University of Pampa—Campus Itaqui, Itaqui 97650-000, RS, Brazil; 3 Research Group on Biochemistry and Toxicology of Bioactive Compounds (GBTOXBIO), Federal University of Pampa—Campus Uruguaiana, Uruguaiana 97500-970, RS, Brazil; jeanboldori.aluno@unipampa.edu.br (J.R.B.); cristianedenardin@unipampa.edu.br (C.C.D.); 4 Department of Pharmacy, State University of Centro-Oeste—Campus Cedeteg, Guarapuava 85040-167, PR, Brazil; tuanebs@gmail.com

**Keywords:** Parkinson’s disease, apoptotic, Lewy bodies, Brazilian purple cherry, p-53/Bax/Bcl-2, intranasal MPTP

## Abstract

**Background:** Parkinson’s disease (PD) is a neurodegenerative disorder marked by motor deficits and non-motor symptoms, such as depression, which are associated with dopaminergic loss and α-synuclein aggregation in the brain. **Objectives:** This study investigated the neuroprotective effects of a hydroalcoholic extract of the purple fruit of *Eugenia uniflora* (PFEU) on motor ability and depressive-like behaviors in a PD model induced by 1-methyl-4-phenyl-1,2,3,6-tetrahydropyridine (MPTP) in female Wistar rats. **Methods:** Rats received intranasal administration of MPTP or vehicle, followed by 14 days of oral administration of PFEU (300 or 2000 mg/kg, administered once daily) or vehicle. Depressive-like behavior was assessed using the splash and forced swimming tests, while motor ability was evaluated using the rotarod and open field tests. On day 15, hippocampal tissue was collected for immunoreactivity analysis. **Results:** MPTP treatment induced depressive-like behavior, which was significantly reversed by PFEU, as evidenced by increased grooming and decreased immobility. No motor coordination or locomotion deficits were observed. Furthermore, PFEU treatment prevented the MPTP-induced increase in hippocampal α-synuclein, p-p53, and Bax while restoring Bcl-2 levels, suggesting neuroprotective effects through the modulation of apoptotic pathways and α-synuclein. **Conclusions:** These findings support PFEU’s potential as a neuroprotective agent for MPTP-induced depressive-like behavior in female rats, highlighting its molecular mechanisms.

## 1. Introduction

The exploration of nutraceutical foods for medicinal purposes has gained momentum in recent research endeavors. In this context, the Myrtaceae family stands out with a variety of noteworthy species. Among them is *Eugenia uniflora*, a tree species distinguished by its dense foliage and primarily found in Brazil’s southern and southeastern regions [1,2]. Its fruit, colloquially known as *pitanga* or Brazilian cherry, is recognized for its flavorful berries, characterized by 8–10 distinctive longitudinal grooves on the skin. In Tupi (indigenous Tupi language), *pitanga* translates to red—a typical fruit hue, although yellow and purple varieties also exist [2].

Brazilian cherries are abundant in magnesium, phosphorus, calcium, potassium, vitamin C, anthocyanins, flavonols, and carotenoids [1]. Of note, the purple fruit variety of *Eugenia uniflora* is the anthocyanin-richest variety, with unparalleled antioxidant properties [3,4]. Anthocyanins are predominant in purple-hued fruits, such as blackberries and Brazilian purple cherries, and can cross the blood-brain barrier and reach brain regions essential for cognitive and emotional functions [5,6,7].

Our research group has been at the forefront of discoveries regarding the neuroprotective potential of a hydroalcoholic extract derived from the purple fruit of *Eugenia uniflora* (PFEU) in a rodent model of Parkinson’s disease (PD) [3,4]. PFEU has demonstrated the ability to mitigate impairments in social recognition memory, short- and long-term object recognition, and working memory in male rats treated with 1-methyl-4-phenyl-1,2,3,6-tetrahydropyridine (MPTP), primarily by modulating hippocampal synaptic transmission and plasticity [4]. Furthermore, PFEU has neuroprotective activity against MPTP-induced oxidative stress [3]. However, the PFEU effects on the emotional impairments induced by MPTP have not been evaluated. PFEU effects are largely associated with its various bioactive compounds, particularly its anthocyanin profile, which contains delphinidin-3-O-glucoside, cyanidin-3-O-glucoside, pelargonidin-3-O-glucoside, cyanidin-3-O-pentoside, and cyanidin derivatives [8].

Depressive disorder is a common early non-motor symptom in PD, affecting up to 40% of PD carriers who display anhedonia, sadness, exhaustion, irritability, and feelings of helplessness. Besides the negative impact on quality of life, depressive disturbances still affect other clinical aspects, such as motor disabilities and cognitive deficits [9]. However, depression in PD is frequently treated with antidepressants, and a high proportion of patients who remain undertreated or not are roughly stabilized with medication, indicating the need for novel pharmacological approaches [10].

Intranasal (in) administration of MPTP leads to behavioral impairments (olfactory, emotional, cognitive, and motor deficits) and dopaminergic degeneration in rats and mice, mirroring those observed in PD carriers [11,12]. Early synaptic and plastic changes are dependent on α-synuclein. Nevertheless, abnormal forms of α-synuclein aggregate into neurons, being the major component of Lewy bodies, which is a neuropathological hallmark of PD. Soon, the accumulation, overexpression, or misfolding of α-synuclein increases neurotoxicity, leading to progressive dopaminergic neuronal death in patients with PD [13].

Given the above, the aim of the present study was to investigate the neuropharmacological potential of PFEU on the depressive-like behavior in female rats subjected to a PD model induced by MPTP administration. Additionally, we evaluated the effects of PFEU treatment on hippocampal markers related to apoptotic pathways and α-synuclein levels.

## 2. Materials and Methods

### 2.1. Animals

The study utilized female Wistar rats weighing 250–300 g and approximately three months old. The animals were obtained from the Federal University of Pampa, Brazil, and handled following the ethical guidelines approved by the Committee on Care and Use of Experimental Animal Resources at the Federal University of Pampa, Brazil (protocol #034/2022). Every possible effort was exerted to minimize any potential discomfort to the animals and to optimize the judicious use of experimental subjects; the animals were kept in cages with 3–4 individuals per unit. The rats were housed under conditions maintaining a temperature range of 22–25 °C, with free access to food and water, and subjected to an artificial 12:12 h light/dark cycle with the onset of illumination at 7:00 a.m. All procedures were conducted between 8:00 a.m. and 4:00 p.m.

### 2.2. Preparation and Characterization of the Hydroalcoholic Extract of Brazilian Purple Cherry

The ripe fruits of *Eugenia uniflora*, botanically identified, were cultivated and collected by the Brazilian Agricultural Research Corporation (Empresa Brasileira de Pesquisa Agropecuária—Embrapa, Pelotas, RS, Brazil—Coordinates: latitude 31°42′ S, longitude 52°24′ W. Altitude: 57 m). The hydroalcoholic extract of Brazilian purple cherry (PFEU) was prepared as described by Savall and collaborators [4] and Tambara and collaborators [8] from the ripe fruits of *Eugenia uniflora*. Total phenolic compounds were quantified using the Folin-Ciocalteu method, with a chlorogenic acid curve serving as the standard (purity 95%; Sigma-Aldrich, St. Louis, MO, USA) [14]. The PFEU exhibited a total phenolic content of 11,252.56 mg chlorogenic acid equivalent per milliliter (CAE/mL). The purple pitanga extract was previously characterized by HPLC-MS/MS by a partner research group [8]. The extract contains the following anthocyanins: cyanidin 3-O-glucoside (512.01 mg/100 g fruit); delphinidin 3-O-glucoside (99.65 mg/100 g fruit); pelargonidin 3-O-glucoside (2.16 mg/100 g fruit); cyanindin 3-O-pentoside (0.83 mg/100 g fruit) and cyanidin derivative (5.16 mg/100 g fruit). In addition, the extract contained 27 phenolic compounds, including two anthocyanins, 20 non-anthocyanin phenolic compounds, and 5 non-identified compounds. All 20 non-anthocyanin phenolic compounds found were derived from five aglycones: quinic acid, gallic acid, HHDP (hexahydroxydiphenyl), myricetin, and quercetin.

### 2.3. Experimental Design and Treatments

The rats were randomly allocated into five groups (n = 11–14 animals/group): (i) control: saline (in) + distilled water (ig); (ii) MPTP: MPTP (in) + distilled water (ig). (iii) PFEU 2000: saline (in) + PFEU 2000 mg/kg/day (ig); (iv) MPTP + PFEU 300: MPTP (in) + PFEU 300 mg/kg/day (ig), (v) MPTP + PFEU 2000: MPTP (in) + PFEU 2000 mg/kg/day (ig). After that, rats were anesthetized with 0.96% isoflurane (0.75 minimum alveolar concentration; Abbott Laboratórios do Brasil, Rio de Janeiro, RJ, Brazil) and the intranasal administration of MPTP HCl (Sigma-Aldrich, St. Louis, MO, USA) or saline, following a protocol outlined elsewhere [4,12]. A 10-mm PE-50 tube was inserted into the nostrils and connected to a peristaltic pump set to deliver at a flow rate of 10 μL/min. MPTP HCl, prepared at a concentration of 100 mg/mL in 0.9% NaCl (saline), was administered intranasally at a dose of 1 mg per nostril over 1 min.

**Figure 1:**
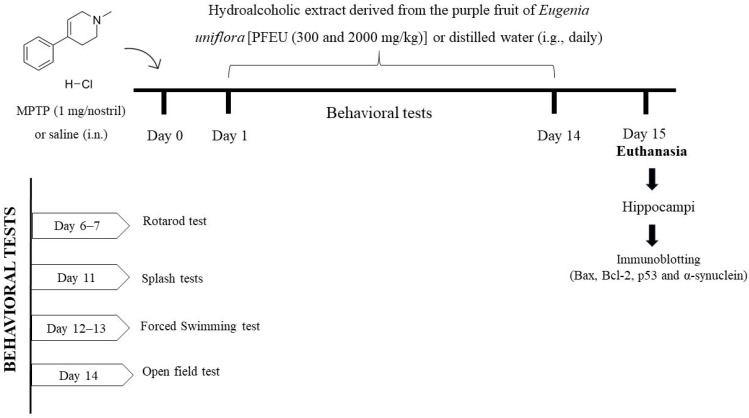
Overview of the experimental protocol. Female rats were administered intranasal MPTP (1 mg/nostril) or saline (on day 0). From day 1 to day 14, they were treated with either PFEU (300 or 2000 mg/kg/day) or distilled water (3 mL/kg/day, orally). Behavioral assessments to evaluate cognitive function were performed between days 6 and 14. On day 15, hippocampal tissue was collected for immunoblotting analysis.

One day after MPTP administration, PFEU treatment began and persisted for 14 days through intragastric gavage (ig). Animals received PFEU at doses of 300 and 2000 mg CAE/kg/day or vehicle (distilled water, 3 mL/kg/day) [4]. The administration of PFEU occurred either after the completion of the behavioral tasks or at the end of the day. Behavioral tests began six days after MPTP administration, and the hippocampi were dissected as whole structures on day 15, as shown in Figure 1.

### 2.4. Behavioral Tests

For animal habituation before any task, the rats remained in the behavior room for at least one hour before the start of activities. The equipment was meticulously cleaned with 20% ethanol between each training and/or testing session. The behavioral assessments sequentially included rotarod, splash, forced swimming, and open field tests (Figure 1). The behavioral tests were conducted by blind observers.

#### 2.4.1. Rotarod Test

The rotarod apparatus comprises a rod (diameter: 8 cm, length: 30 cm) divided into four sections by 10 cm diameter discs (Insight, Ribeirão Preto, Brazil). The rod rotates steadily at 10 rpm. Rats underwent a preliminary training session (day 6), with each rat placed on the rod until it mastered the task. On the testing day (day 7), the rats were positioned atop the rotating beam, and the time until their first fall was recorded by an observer blinded to the experiment (cut-off: 300 s). This procedure consisted of three trials spaced 10 min apart [15].

#### 2.4.2. Splash Test

The splash test, conducted on the 11th day, involves applying a 10% sucrose solution to a rat’s back, which induces grooming behavior [16]. Grooming activity is then observed for 5 min, indicating self-care and motivation levels. This test is utilized to assess motivational behaviors that are thought to mirror certain aspects of depression, such as behaviors indicative of apathy.

#### 2.4.3. Forced Swimming Test

The forced swimming test was performed following the protocol by Porsolt et al. [17], with minor adjustments. Rats were individually placed in open plastic cylinders (40 cm high, 30 cm in diameter) containing 25 cm of water and maintained at 25 ± 1 °C. A pretest session was conducted 12 h before treatment (day 12), during which the rats swam for 15 min before being returned to their home cages. The test session occurred 24 h after the pretest (or 12 h post-treatment, on day 13), during which the rats underwent the forced swimming test again, and immobility time was recorded for 5 min. Immobility was defined as rats floating passively in the water, making only the minimal movements required to keep their heads above the surface.

#### 2.4.4. Open Field Test

The open field floor was partitioned into nine squares. Each rat was placed individually in the center of the arena, and their activity was assessed based on the number of segments crossed (four-paw criterion) and rearings, which were recorded during a 5 min session [18]. Locomotor activity was evaluated on day 14 after MPTP administration.

### 2.5. Immunoreactivity

Western blot assay was performed as previously described by Guerra et al. [19]. Hippocampus samples were manually homogenized in a microtube containing 300 µL of ice-cold buffer A (10 mM KCl, 2 mM MgCl_2_, 1 mM NaF, 1 mM EDTA, 10 µg/mL aprotinin, 10 mM β-glycerolphosphate, 1 mM PMSF, 1 mM DTT, and 2 mM sodium orthovanadate in 10 mM HEPES, pH 7.9), incubated for 15 min at 0 °C, and then centrifuged at 4 °C for 45 min at 16,000× *g*. The supernatant was collected, and the protein concentration was determined using equivalent amounts of protein (80 µg). Subsequently, 0.2 volumes of concentrated loading buffer (200 mM Tris, 10% glycerol, 2% sodium dodecyl sulfate (SDS), 2.75 mM β-mercaptoethanol, and 0.04% bromophenol blue) were added to the samples, which were then boiled for 5 min.

During the electrophoresis process, proteins were separated using a 12% SDS-polyacrylamide gel (SDS-PAGE) for approximately 3 h and transferred to a nitrocellulose membrane using the Transfer-Blot Turbo Transfer System (1.0 mA; 30 min). To confirm successful transfer, the membranes were stained with a 0.5% Ponceau solution. After blocking using 1% bovine serum albumin solution, the blots were incubated overnight at 4 °C with specific primary antibodies, including mouse anti-α-synuclein antibody (1:1000, Santa Cruz Biotechnology, Santa Cruz, CA, USA, code sc-515879), mouse anti-Bax (1:500, Santa Cruz Biotechnology, Santa Cruz, CA, USA, code sc-7480), mouse anti-Bcl-2 (1:500, Santa Cruz Biotechnology, Santa Cruz, CA, USA, code sc-7382), and rabbit anti-phospho-p53 (p-p53) (1:1000, Sigma-Aldrich, St. Louis, MO, USA, code SAB4503953).

The membranes were washed and incubated with secondary antibodies conjugated with horseradish peroxidase (1:5000, anti-rabbit IgG or anti-mouse IgG-HRP; Santa Cruz Biotechnology, Santa Cruz, CA, USA) for 2 h. Detection was achieved using 3,3′,5,5′-tetramethylbenzidine (Sigma-Aldrich, St. Louis, MO, USA), and ImageJ software 1.54g (NIH, Bethesda, MD, USA) was used for quantification of the optical density of the immunoblotting bands. The results were normalized by arbitrarily setting the densitometry of the control group as 100%, based on the ratio between the protein band and the 0.5% ponceau load control, which serves as a validated alternative to actin in Western blots [20].

### 2.6. Statistical Analysis

Statistical analyses were carried out using one-way analysis of variance (ANOVA), followed by Tukey’s multiple range test when applicable. The statistical analyses were conducted using GraphPad 8 software (San Diego, CA, USA). In the case of the one-trial ORT, a one-sample t-test was utilized to compare the recognition percentage with the theoretical value of 50%. All results are reported as mean ± standard error of the mean (SEM), and statistical significance was defined at *p*-values < 0.05 for all analyses.

## 3. Results

### 3.1. PFEU Protected Against Depressive and Anhedonic-like Behavior Induced by MPTP in Female Rats

**Figure 2:**
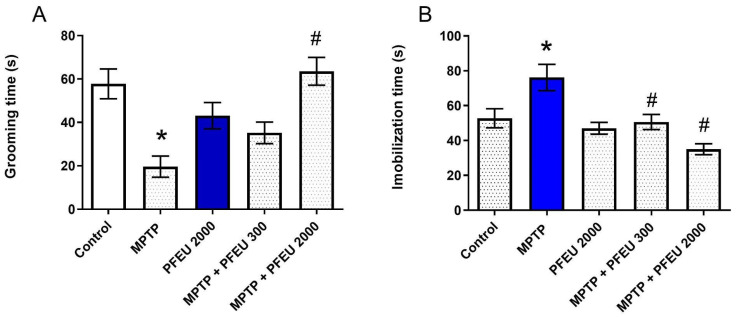
Effects of PFEU on depressive-like behaviors in female rats treated with intranasal MPTP. Behavioral outcomes in (**A**) the Splash test and (**B**) the Swimming test were assessed following treatment with PFEU at doses of 300 and 2000 mg/kg/day in the context of MPTP administration. Results are expressed as the mean ± SEM (n = 8–11 per group). * Indicates *p* < 0.05 versus the control group, and # indicates *p* < 0.05 versus the MPTP group (one-way ANOVA with Tukey’s post-hoc test).

Regarding anhedonia-like behavior, one-way ANOVA showed a significant difference among the groups in self-care time (grooming) in the splash test (F_(4,44)_ = 9.886; *p* < 0.0001; Figure 2A). Tukey’s post-hoc test corroborated that MPTP-treated animals displayed a reduction in grooming time compared to the control group (*p* = 0.0004). Importantly, PFEU treatment at a dose of 2000 mg/kg/day blocked this effect (*p* < 0.0001; Figure 2A).

One-way ANOVA analysis of immobility time in the forced swimming test indicated a significant difference among the experimental groups (F_(4,44)_ = 9.206; *p* < 0.0001; Figure 2B). Subsequent post-hoc analysis revealed that the MPTP-treated group exhibited a notable increase in immobility time compared to the control group (*p* = 0.0273). Both doses of PFEU blocked the depressive-like effect caused by MPTP in a dose-dependent manner, as observed through a reduction in the total immobility time (*p* = 0.0055 and *p* < 0.0001, respectively).

### 3.2. Motor Changes Were Not Observed in Both Rats Exposed to MPTP and Those to PFEU

One-way ANOVA revealed no significant difference among the experimental groups in motor coordination assessed in the rotorad test (F_(4,44)_ = 0.8854; *p* = 0.4806) or in the crossing number (F_(4,44)_ = 2.194; *p* = 0.0852) measured in the open field test (Table 1). One-way ANOVA showed a significant difference in the number of rearings (F_(4,44)_ = 2.987; *p* = 0.0289) measured in the open field test. However, no significant differences between the groups were found in the subsequent Tukey’s post-hoc test (Table 1).

### 3.3. PFEU Treatment Effectively Suppressed the Elevation of α-Synuclein Levels and Inhibited the Activation of the Apoptotic Pathway Induced by MPTP

**Figure 3:**
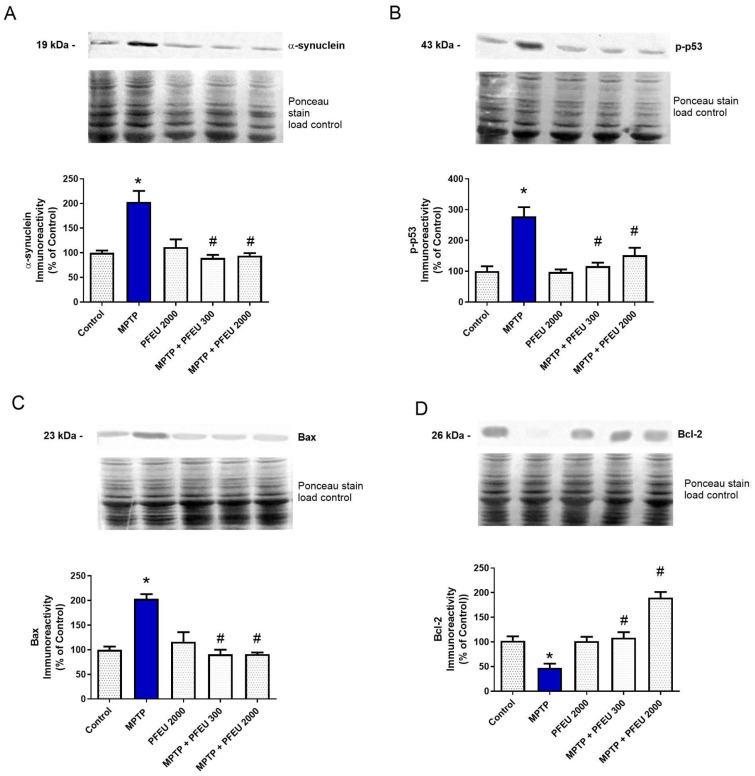
Effects of PFEU on hippocampal apoptotic markers and α-synuclein levels in female rats treated with intranasal MPTP. Hippocampal protein levels of (**A**) α-synuclein, (**B**) p-p53, (**C**) Bax, and (**D**) Bcl-2 were analyzed following treatment with PFEU at doses of 300 and 2000 mg/kg/day in the context of MPTP administration. Results are expressed as the mean ± SEM (n = 5/group). * Indicates *p* < 0.05 versus the control group, and # indicates *p* < 0.05 versus the MPTP group (one-way ANOVA with Tukey’s post-hoc test).

To investigate the possible mechanisms underlying the observed effects of PFEU, immunoblotting assays were conducted on the hippocampi of the animals (Figure 3). One-way ANOVA revealed notable differences in hippocampal α-synuclein levels among the groups (F_(4,19)_ = 13.01, *p* < 0.0001; Figure 3A). Post-hoc analysis demonstrated that MPTP administration increased α-synuclein levels (*p* = 0.0002), while PFEU treatments effectively blocked these changes (MPTP + PFEU 300, *p* < 0.0001 and MPTP + PFEU 2000, *p* = 0.0001).

Significant differences in hippocampal p-p53 levels among the groups were revealed by one-way ANOVA (F_(4,19)_ = 14.38, *p* < 0.0001; Figure 3B). Post-hoc analysis indicated a substantial increase in p-p53 levels in the MPTP group (*p* < 0.0001); however, no differences were observed between the control group and MPTP + PFEU 300 and MPTP + PFEU 2000 groups, indicating that PFEU treatment prevented the increase in p-p53 levels (MPTP + PFEU 300 and MPTP + PFEU 2000 differed significantly from MPTP, with *p*-values of 0.0001 and 0.0016, respectively).

One-way ANOVA also demonstrated significant differences in hippocampal Bax (F_(4,19)_ = 23.75, *p* < 0.0001; Figure 3C) and Bcl-2 (F_(4,19)_ = 24.70, *p* < 0.0001; Figure 3D) levels among the groups. Post-hoc analysis indicated that MPTP administration resulted in significant upregulation of Bax levels (*p* < 0.0001) and downregulation of Bcl-2 levels (*p* = 0.0055). Conversely, PFEU treatment effectively attenuated these changes, with both the MPTP + PFEU 300 and MPTP + PFEU 2000 groups exhibiting substantial reductions in Bax levels (*p* < 0.0001) and increases in Bcl-2 levels (*p* = 0.0020 and *p* < 0.0001, respectively).

## 4. Discussion

Our data demonstrate the remarkable neuroprotective effect of PFEU against anhedonia-like and depressive-like behaviors induced by MPTP in female rats. The neuroprotective effects of PFEU are partly attributed to the modulation of the p-p53/Bax/Bcl-2 signaling pathway, along with its influence on α-synuclein levels in the hippocampus.

MPTP is a pro-neurotoxin that crosses the blood-brain barrier, being metabolized by monoamine oxidase B (MAO-B) into 1-methyl-4-phenylpyridinium, a toxic molecule in glial cells. Thereafter, it is transported by dopamine transporters to neurons, where it inhibits mitochondrial complex I, leading to neurodegeneration [12,21]. The MPTP-based model reliably causes lesions in the nigrostriatal pathway and mimics the neuropathological and clinical features of PD observed in humans. These features include cognitive decline, depression, sleep disturbances, and olfactory dysfunction in mammals such as rats and monkeys [21,22]. Moreover, the olfactory system serves as a route for toxic agents to reach the central nervous system. MPTP has been shown to cause olfactory, cognitive, emotional, and motor alterations in rodents [12,21,22].

Depression can appear decades before the diagnosis of PD, indicating that it is a possible early marker of the disease [23]. In animals, studies have shown that exposure to MPTP alters genes related to apoptosis and that mood disorders share pathogenic mechanisms with PD [24]. Other studies have shown that MPTP induces depressive-like behavior, which has been observed during the prodromal period in rodents using various methods of administration, including intraperitoneal and intranasal routes [25]. In this study, we also observed that rats exposed to MPTP exhibited depressive-like behavior, which was mitigated by PFEU; this behavior was evident in both the forced swimming and splash tests.

The main symptom of PD is the presence of motor abnormalities, although cognitive and emotional dysfunctions are prevalent non-motor symptoms in early PD [26,27]. Herein, no motor coordination or locomotor impairment was observed in any of the tested groups, as evidenced in the rotarod and open field tests, thus confirming that PFEU promotes antidepressant-like behavior without affecting motor skills. However, using the same experimental design, MPTP decreased locomotor activity in male rats [4]. Corroborating these results, Antzoulatos et al. [28] observed distinct differences between male and female mice after MPTP administration, with only males exhibiting decreased stride length. These findings further support the hypothesis that MPTP induces sexually dimorphic impairments in motor performance. Moreover, Schamne et al. (2018) also reported that intranasal MPTP administration induces more pronounced anhedonia and selective depressive-like behaviors in female adult mice, with similar effects observed in ovariectomized (OVX) and aged female mice. The gender-related differences in emotional responses to MPTP appear to be associated with a greater depletion of neurotrophins, particularly BDNF and GDNF, in the hippocampus and prefrontal cortex of female mice [25].

In rats, anhedonia is commonly assessed through behavioral tests that evaluate hedonic (pleasure-seeking) behavior, which is often impaired in conditions such as depression. One widely used method for this purpose is the splash test [29,30] (Mutlu et al., 2012; Isigrini et al., 2010). This test measures the decrease in self-grooming or licking behavior in response to a sucrose solution, with a neuroprotective effect indicated by the restoration of normal licking behavior following treatment with PFEU. Experimental models have successfully replicated the central non-motor symptoms of PD, including anhedonia and depression-related defensive behaviors. The splash test serves as a reliable indicator of anhedonic behavior, as demonstrated by Matheus et al. (2016) [31] and Marques et al. (2019) [32]. Furthermore, it has been effective in detecting anhedonia in MPTP-induced models, particularly in female rodents, as shown by Moretti et al. (2015) [33] and Schamne et al. (2018) [25].

PD affects men twice as often as women, although there is a faster mortality rate and progression of the disease in women, especially after the onset of menopause [27,34]. Despite this, sex differences in PD symptoms and treatment responses have often been neglected, despite evidence suggesting the need for sex-specific approaches [34,35]. Existing literature on memory, depression, and biological sex in PD is limited and challenging to understand [35]. Gender influences depression [36,37,38] and memory deficits [35,39,40]. Women with PD often have better verbal memory, while men show superior visuospatial skills; these differences lessen as the disease progresses [41]. Non-motor symptoms in women emerge early, marking specific sex differences [42]. Depression is more prevalent in women and is linked to disease severity [37,38]. Generally, women have lower physical function and socioemotional health scores, while men experience faster cognitive decline [37]. In this context, this study also contributes to presenting an effective approach against depression-like behavior in females.

Changes in normal α-synuclein function related to neuropsychiatric symptoms in PD remain largely unclear [43,44,45,46]. In the context of PD, fibrillar α-synuclein can prompt the formation of pathological inclusions resembling Lewy neurites and Lewy bodies from normal α-synuclein. These aggregates corrupt endogenously expressed α-synuclein, spread throughout the brain, and affect presynaptic regions in the dentate, hilar, and CA2/3 regions of the hippocampus in human brains with PD [47,48,49]. Therefore, addressing α-synuclein aggregates in a prodromal state is essential for developing therapies to prevent the onset of more severe symptoms. For instance, Hijaz and Volpicelli-Daley [47] suggested that α-synuclein overexpression and deposition in cell bodies may be directly linked to neurodegeneration in MPTP-exposed non-human primates. Indeed, increased α-synuclein gene expression after MPTP exposure and neuronal death have been observed in rodents, and varying degrees of neuronal dysfunction and degeneration have been found in vitro [37,38,44,48].

Our results showed that MPTP administration increased hippocampal levels of α-synuclein, suggesting a possible increase in the aggregation of this protein in the rat brain. Similarly, McCormack et al. [50] demonstrated that a single injection of the neurotoxin MPTP in squirrel monkeys led to the accumulation of α-synuclein in nigral dopaminergic cell bodies. This indicates that the toxic lesion may induce modifications of α-synuclein implicated in the pathogenesis of human synucleinopathies, with the accumulation and aggregation of the protein in damaged axons.

Furthermore, various studies have demonstrated increased α-synuclein levels in both rodents and monkeys treated with MPTP. These studies showed an association between elevated α-synuclein levels and decreased neuronal cells in different brain regions, including the hippocampus [45,50,51]. Consistent with this, our findings revealed that both doses of PFEU were effective in reducing hippocampal α-synuclein levels. Similar observations were made by Maulik et al. [52], who used blueberry extract and attributed the neuroprotective effects to the composition of anthocyanin-rich extract and proanthocyanidin, which also conferred protection against oxidative stress in vitro and extended shelf life in a *C. elegans* model.

Furthermore, our data align with those of previous studies, indicating that MPTP induces p53 phosphorylation. Activation of p53 induces Bax expression and promotes permeabilization of the outer mitochondrial membrane, contributing significantly to neuronal death in neurodegenerative diseases [53]. In this sense, p-p53 plays a transcriptional regulatory role by activating pro-apoptotic genes, while suppressing the expression of anti-apoptotic genes from the Bcl-2 family. This mediation is executed by Bax and PUMA (p53-upregulated mediator of apoptosis), representing a critical link between p53 and Bax in neurodegenerative diseases [53,54].

Notably, PFEU treatment can mitigate the damage caused by MPTP and prevent the initiation of the apoptotic cell death pathway. Specifically, we observed increased p-p53 and Bax levels in the hippocampi of animals treated with MPTP, indicating the onset of the apoptotic process. In contrast, PFEU treatment blocked these effects at both doses tested. Additionally, Bcl-2 levels, which promote cell survival and proliferation, were decreased by MPTP but increased at 300 and 2000 mg/kg doses of PFEU. Similarly, the Bax/Bcl-2 ratio decreased after treatment with an anthocyanin-containing extract Chen and collaborators [55] associated this modulation with a reduced rate of individual nerve cell apoptosis, thereby slowing memory dysfunction, neuroinflammation, and neurodegeneration in a d-galactose model in rodents.

Although changes in apoptotic markers and increased α-synuclein levels have been observed, additional studies, including the assessment of immunohistological and histopathological alterations in the hippocampus, are needed. Strong validation is essential to substantiate claims regarding cell death, particularly with respect to α-synuclein aggregation. Therefore, caution should be exercised when interpreting these findings. Future research will be crucial to confirm and further support the proposed neuroprotective effect of PFEU.

The neuroprotective effects of *Eugenia* species have been reported in various animal models. For instance, *Eugenia dysenterica* leaves at 100 and 300 mg/kg showed STM- and LTM-related neuroprotective effects, which were linked to their antioxidant capacity [56]. Flores et al. [57] found that chronic administration of an *Eugenia uniflora* fruit (red type) extract exhibited an antidepressant effect by regulating acetylcholinesterase (AChE) activity and reactive oxygen species production in the prefrontal cortex and hippocampus. Similar results using red fruit-type *Eugenia uniflora* were obtained by Oliveira et al. [58], who observed benefits against metabolic alterations and antioxidant and antidepressant effects by improving AChE activity in the hippocampus and prefrontal cortex. These effects were attributed to the presence of nine anthocyanins as the main compounds in the fruit. The primary anthocyanin found in Brazilian purple cherry is cyanidin-3-O-glucoside, followed by delphinidin-3-O-glucoside, accounting for 82.7 and 16.1% of the total anthocyanin content in PFEU extract, respectively, but other bioactive compounds, such as myricetin and gallic acid, were found in the extract [8]. Hence, we believe that the neuroprotective effect of PFEU, mainly its potential against MPTP-induced damage when administered intranasally in female rats, is due to the multiple compounds present in the extract.

## 5. Conclusions

This study reveals the therapeutic potential of the fruit of *Eugenia uniflora*, specifically its hydroalcoholic extract (PFEU), in offering neuroprotection against anhedonic and depressive-like behavior in a PD model induced by MPTP administration in female rats. Our results indicate that the apoptotic pathway plays a significant role in the neuronal damage caused by MPTP and that PFEU effectively mitigates this damage. Moreover, PFEU significantly reduced hippocampal α-synuclein levels and pro-apoptotic markers that were elevated due to MPTP exposure. These findings suggest that one of the mechanisms through which PFEU improves emotional behavior in this PD model may involve its modulatory effects on neurochemical markers. Thus, our study reports the potential neuroprotective effect of *Eugenia uniflora* against a model of PD in female rodents, emphasizing the importance of further research to elucidate this relationship, especially by evaluating other apoptotic markers necessary for elucidating the effect of this extract.

## Figures and Tables

**Table 1:** Groups did not differ in the total number of crossings and rearings in the open field and rotarod tests.

	Crossing Number	Rearing Number	Elapsed Time (s)
Control	54.25 ± 6.67	21.38 ± 2.66	292.5 ± 7.500
MPTP	49.46 ± 4.07	20.27 ± 1.88	300 ± 0.000
PFEU 2000	40.75 ± 3.02	14.63 ± 1.67	292.5 ± 7.500
MPTP + PFEU 300	36.90 ± 4.62	13.36 ± 2.12	298.2 ± 1.818
MPTP + PFEU 2000	49.91 ± 5.32	14.91 ± 1.96	300 ± 0.000

The number of crossings and rearings in the open field test and performance in the rotarod test were evaluated following treatment with PFEU at doses of 300 and 2000 mg/kg/day in the context of MPTP administration. Results are expressed as mean ± SEM (n = 8–11/group).

## Data Availability

The complete dataset supporting the obtained results is included in the article, and there is no need for supplementary source data.
